# A Poor Half-Witted Girl

**Published:** 1887-01-08

**Authors:** J. Wilson

**Affiliations:** Hon. Sec. Workhouse Infirmary Nursing Association, 44, Berners Street, W.


### Difficult Cases.

A Poor Half-witted Girl.

Who, of all our readers, will lend a hand to help this deserving and interesting case?—

Can you or your readers kindly give some advice regarding the following "Difficult case." It is that of a half-witted girl of about fifteen who is in the small workhouse of a country town. She cannot, of course, get her own living in service, and though she can read a little, sew a little, and do simple sums, it seems difficult to know in what way she could be employed, and it seems a pity for her to remain with adults. Are paupers taken at Earlswood, or is there any similar institution for paupers where they can be trained to do needlework or to make baskets, or anything they may be capable of. I should be glad of information as to the probable cost of the same, and if Guardians as a rule are willing to spend on such cases.—I am, yours faithfully,

J. Wilson, Hon. Sec.
Workhouse Infirmary Nursing Association,
44, Berners Street, W.

